# We Need to Protect Ourselves: The Mental Burden of Managing Type 2 Diabetes among Black men During the COVID19 Pandemic

**DOI:** 10.1093/geroni/igab046.2728

**Published:** 2021-12-17

**Authors:** Ledric Sherman, Anthony Pattin, Carla Pattin, Michelle Strong, Sara Vera

**Affiliations:** 1 Texas A&M University, College Station, Texas, United States; 2 The University of Toledo, Toledo, Ohio, United States

## Abstract

As COVID-19 swept across the United States in 2020, it appeared to infect and kill Black Americans at a disproportionately high rate. When examining the literature pertaining to the pandemic, present COVID-19 research focuses on physical health, but research regarding mental health and stress during the COVID-19 pandemic are lacking, especially among Black men and minority men in general. Therefore, the purpose of this study was to learn more about the type 2 diabetes (T2D) management related stress among Black men (n=22) during the COVID-19 pandemic. One on one interviews were conducted via Zoom video conferencing to gain an understanding of the experiences of managing T2D in a pandemic environment. Three main themes emerged from the study, which are: (1) my stress levels during this time, (2) diabetes specific stressors, and (3) coping mechanisms. Black men with diabetes may need psychosocial support that holistically addresses general developmental and diabetes-specific stressors and their influences on one another. Recognizing how Black men react to stress is essential for understanding and addressing their disproportionally high prevalence of stress related to management of chronic conditions and avoiding additional complications, morbidity or mortality. It is imperative to examine how stress and coping frameworks relate to men’s health, in general, but specifically in Black men.

